# Overlapping Phenotypes in Osteopetrosis and Pycnodysostosis in Asian-Indians

**DOI:** 10.1155/2021/7133508

**Published:** 2021-11-03

**Authors:** Parminder Kaur, Inusha Panigrahi, Harleen Kaur, Thakurvir Singh, Chakshu Chaudhry

**Affiliations:** Department of Pediatrics, APC, PGIMER, Chandigarh, India

## Abstract

Osteopetrosis is a disorder characterized by high bone density, hepatosplenomegaly, visual and hearing loss, and anemia. Pycnodysostosis presents with short stature, acroosteolysis, and dense bones. We, hereby, present here a family with autosomal dominant osteopetrosis and also children with recessive osteopetrosis and pycnodysostosis. The molecular confirmation was done in 3 cases. Genetic heterogeneity in clinical presentation is discussed here. Further studies will help in identifying epigenetic alterations and population-specific variants and also developing targeted therapies.

## 1. Introduction

Osteopetrosis was first reported by a German radiologist in 1904 and also known as marble bone disease [1]. It is a rare hereditary bone disorder caused due to the failure of normal osteoclast function leading to increasing bone density. The encroachment of the medullary spaces by bones leads to compensatory extramedullary hematopoeisis leading to hepatosplenomegaly and bone marrow failure. The occlusion of intracranial foramina by bone can lead to blindness, deafness, or nerve palsies. Defective bone resorption manifests with dysmorphic features in the form of macrocephaly, hypertelorism, frontal bossing, proptosis, and retarded linear growth with psychomotor delay [2]. Dental anomalies in the form of delayed tooth eruption, retained deciduous teeth, dental caries, and defects of periodontal membrane along with thickened lamina dura may be seen [3]. Osteopetrosis may be inherited as autosomal dominant usually manifesting in adults and is relatively benign form or the autosomal recessive disease which manifests in infants and is mostly malignant form and typically fatal [4]. Severe autosomal recessive osteopetrosis or malignant osteopetrosis occurs due to homozygous or compound heterozygous mutations in multiple genes implicated in abnormal bone modelling and causing increased bone density. Milder forms of osteopetrosis were earlier reported to be mostly autosomal dominant in inheritance with later presentation in adolescence or adulthood. However, with more mutations being identified, milder forms of recessive disorder are also being recognized. Another disorder of increased bone density presenting with short stature as a predominant manifestation is pycnodysostosis. Recently variants in the *CTSK* gene have also been reported in patients with intermediate osteopetrosis [5]. The X-rays in osteopetrosis reveal dense bones evident in the skull, spine, the pelvis, and appendicular bones, along with cortical thickening and medullary encroachment. The paranasal sinuses are poorly visualised, and sandwich appearance of vertebrae and bone in bone appearance are observed due to sclerosis along with radiolucent transverse metaphyseal lines [6]. Treatment for osteopetrosis is symptomatic, and the only curative treatment is bone marrow stem cell transplantation (BMSCT) [7]. We report the phenotypes of some children with osteopetrosis and pycnodysostosis who were seen in the outpatient clinic or admitted in the ward for detailed evaluation and management.

## 2. Case Reports

We had 7 cases of osteopetrosis/pycnodysostosis from 5 families in last 5 years seen in the genetic metabolic unit. This included 1 familial osteopetrosis and 3 isolated patients. 3 patients were from Jammu and Kashmir state.

### 2.1. Cases 1–3

**Figure 1:**
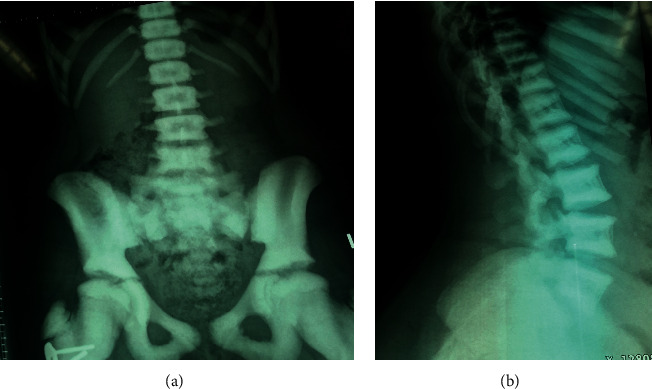
X-ray spine and pelvis showing dense vertebrae and pelvic bones (a), and Sandwich vertebrae (b).

In the family with autosomal dominant osteopetrosis, the mother and 2 daughters were affected. The older daughter was referred for suspected bone disease since she had a fracture for which rod was inserted in lower limb. There was no history of any visual loss. Examination did not reveal any hepatosplenomegaly. Both girls had prominent forehead and deep set eyes. X-rays done in the two daughters showed dense bones were consistent with osteopetrosis (Figure 1). The younger daughter also had hemivertebrae in lumbar spine. The mother's X-rays also revealed dense bones, and hence, a possibility of autosomal dominant osteopetrosis was kept. Mutation testing was done by targeted next generation sequencing in the older daughter and revealed a variant in the exon 10 of the *CLCN7* gene-c.856C > T (R286W). The variant was confirmed in the other daughter and mother by Sanger sequencing.

### 2.2. Case 4

In another family, a 3-year-old girl born out of third degree consanguineous mating was brought to the genetic ward with a complaint of progressive enlargement of head since birth and progressive abdominal distension from 7 months of age. She also had on and off nasal bleed for the last one year. Also, there was a complaint of delayed attainment of milestones, and development quotient (DQ) was 25.

On examination, weight was 6 kilograms (−6.0 *z* score), length was 72 entimeters (−4.11 *z* score) with upper segment to lower segment ratio being 1.18, and head circumference was 49.5 cm (+0.66 *z* score). She had pallor and dysmorphism in the form of large head with frontal prominence, depressed nasal bridge, flat face, low set ears, long philtrum, thin upper lip, oligodontia, Mongolian spots, and brachydactyly. Lumbar kyphosis was also present, along with continuous rotatory nonpurposeful eye movements. There was marked abdominal distension with liver being palpable 3 centimeters below the right subcostal margin, firm in consistency with irregular margins, and spleen was palpable till umbilicus.

**Figure 2:**
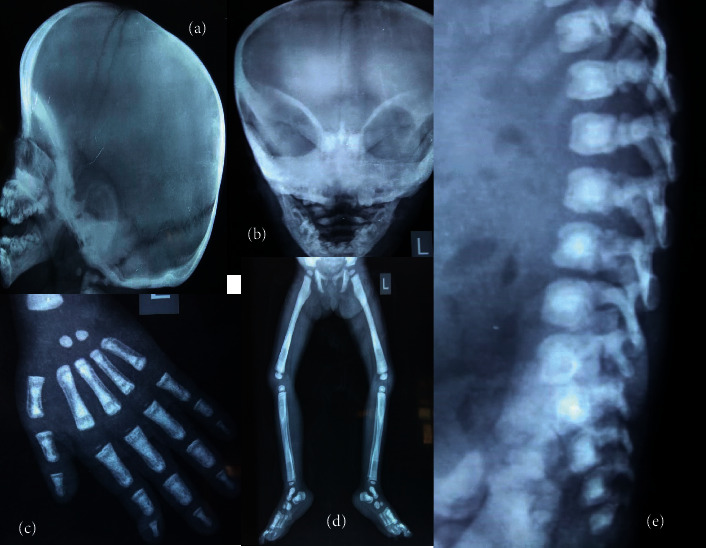
(a) & (b) X-rays in case 4: Skull lateral and AP view showing calvarian and basilar thickening, poorly developed sinuses and sclerosis of nasal bone; (c) X-ray hand showing bone in bone appearance; (d) X-ray lower limbs showing bone in bone appearance and increased bone density; (e) X-ray spine lateral view showing bone in bone appearance; (f) X-ray chest showing increased bone density in ribs and absence of corticomedullary differentiation.

Investigations showed hemoglobin of 7 g/dl and white blood cell (WBC) count of 7000/mm^3^ with 40 percent neutrophils, 45 percent lymphocytes, 4 percent eosinophils, and 8 percent monocytes. The platelet count was 10,000/mm^3^. Renal function and liver function tests and serum electrolytes were normal. Calcium was 8.2 mg/dl, and phosphate was 3.3 mg/dl. Vitamin D level was low (9.12 ng/ml), and parathormone (PTH) level was normal. Skeletal survey showed typical bone in bone appearance in X-rays of hands, long bones, and spine lateral views; skull X-ray showed basilar thickening, nasal bone sclerosis, and poorly formed sinuses; and X-ray of chest showed poor corticomedullary differentiation and increased bone density in ribs (Figure 2). DEXA scan also confirmed increased bone density in spine. Fundus examination revealed bilateral optic atrophy, and visual acuity was limited to bilateral perception of light. Ultrasonography of abdomen showed hepatosplenomegaly with normal echogenicity of kidneys.

Hence, on the basis of radiological findings and as the child was symptomatic in early infancy, the diagnosis of malignant infantile osteopetrosis was made and symptomatic treatment in the form of nutritional rehabilitation and transfusion of platelet concentrates was given, and parents were counseled for BMSCT. Molecular testing could not be done as parents were not ready for the test.

### 2.3. Case 5

**Figure 3:**
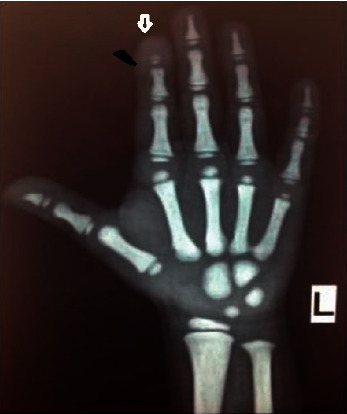
The X-ray of one hand with wrist in case 5-pycnodysostosis patient showing delayed bone age and acroosteolysis in the distal phalanges of thumb and index finger.

A six-year-old female child was brought to the genetic clinic with complaints of poor postnatal growth. Examination revealed short stature, mild dysmorphic features like round face and low set large ears, and short index finger in the hands, but no hepatosplenomegaly. X-ray of the hands revealed increased density of the bones, acroosteolysis in distal phalanges especially in the index finger and thumb and delayed bone age (Figure 3). A possibility of pyknodysostosis was kept, and Sanger sequencing was performed by amplifying exon 1–8 of the *CTSK* gene. A novel heterozygous likely pathogenic variant was found in gene *CTSK*: c.866_866delA: p. (Asn289Thrfs *∗* 5). A second mutation could not be identified, as further testing could not be done due to financial constraints.

### 2.4. Case 6

A male child of 1 year and 8 months presented with swelling over abdomen from 6 months of age and fever for previous 1 month; fever subsided on antipyretics and was associated with irritability. He had a history of blood transfusion 1 year earlier. Examination showed blue sclera, open anterior fontanelle, and enlarged liver and spleen. The weight was 8.7 kg (−2.48 *z*), height was 73 cm (−4.7 *z*), and head circumferences was 46 cm (−1.35 *z*). The liver was enlarged being 5 cm below the right costal margin, and spleen was 7 cm below the left costal margin. Investigation showed pancytopenia with Hb of 9.9 g/dl, the WBC count of 8970/mm^3^, and platelets of 63000/mm^3^. The alkaline phosphatase level was 316 units, and C-reactive protein (CRP) was 30 units. The values of serum transaminases, coagulogram, renal and liver function tests were within normal ranges. Ophthalmological evaluation did not reveal any cherry red spot. Skeletal survey done showed increased bone density in the limbs and spine. Hearing evaluation revealed bilateral type B tympanogram. He was given antibiotics—ceftriaxone and azithromycin—as he had high grade fever. Targeted exome sequencing was done in suspect for disorders with increased bone density like osteopetrosis and pyknodysostosis. The genes covered in the panel were *ClCN7, CTSK, LRP4, LRP5, OSTM1, PTH1R, SNX10, SLC29A3, TCIRG1, TNFSF11, TNFRSF11A, TNFRSF11B,* and *SOST* genes. A likely pathogenic variant was identified in the exon 3 of *CTSK* gene in the homozygous state. The variant was *CTSK:* c.136C > T:p. (Arg46Trp), which was not present in the 1000 genome database, and found to be deleterious on bioinformatic tools including FATHMM, LRT, Mutation Taster, and Polyphen2.

### 2.5. Case 7

A 7-month-old male, born out of consanguineous marriage, presented with developmental delay and pallor. Examination revealed mild dysmorphism and hepatosplenomegaly. Investigations showed thrombocytopenia in addition to anemia and increased bone density with bone in bone. Appearance of vertebral bodies was confirmed on X-rays of the spine and hands. Keeping a possibility of osteopetrosis, a targeted NGS was performed which revealed a homozygous variant in the exon 7 of the *TCIRG1* gene. The variant was *TCIRG1*:c.674G > A:p. (Gly225Asp). The variant has not been reported earlier in 1000 genomes and gnomAD databases, and it is predicted to be damaging on SIFT, LRT, and Mutation Taster 2. The child was counseled regarding disease, need for BMSCT, and prenatal diagnosis in next pregnancy.

## 3. Discussion

Osteopetrosis is a metabolic bone disease caused by failure of osteoclasts leading to impaired bone resorption [8]. The infantile form of osteopetrosis presents at birth or in infancy [9]. Our 4th and 6^th^ case also started having manifestations within first year of life. The abnormal bone replaces the medullary cavity leading to bone marrow failure and extramedullary hematopoiesis causing hepatomegaly and splenomegaly. Also, bony encroachment occurring on the optic foramina may lead to visual loss and optic atrophy [10]. The early onset osteopetrosis cases in current report also presented with features of bone marrow failure in form of anemia, thrombocytopenia, and extramedullary hematopoiesis in the form of hepatosplenomegaly. Also, due to optic nerve entrapment, there was optic atrophy and visual impairment in the form of only light perception in both eyes. The poor development of dental structures in osteopetrosis may be caused by defective osteoclast in this condition, hence leading to failure of alveolar resorption; the other etiology implicated is decrease in the supply of nutrients to the developing tooth [11]. The fourth case described here also had tooth anomalies in form of oligodontia and hypodontia.

Diagnosis is made on radiological findings which show increased bone density with abnormal metaphyseal modelling. The “bone within bone” appearance is diagnostic [9]. Our cases had all these findings on radiology, and the diagnosis was made on clinical features and radiological findings. The definitive treatment of severe osteopetrosis is BMSCT, and the 5-year survival of HLA-matched transplants has been estimated to be 79% [10].

Molecular diagnosis is nowadays feasible by next generation sequencing (NGS). Genes implicated in osteopetrosis include *LRP5, CLCN7, OSTM1, TCIRG1, SNX10, PLEKHM1, TNFRSF11A,* and *TNFSF11.* The first two are usually implicated in milder forms of disease [12]. *TCIRG1, SNX10*, and *TNFSF11* are associated with autosomal recessive early onset form [13]. In our first case with autosomal dominant osteopetrosis mutation in *CLCN7* gene, c.856C > T was found, a likely pathogenic variant. This has been reported in an earlier study [14]. Compound heterozygous variants in *CLCN7* are interestingly known to cause autosomal recessive osteopetrosis. A 15-year-old female with IARO was described earlier who had onset of features at 5 years of age with fractures, dense ones, hypodontia, and mild visual loss due to optic atrophy by Okamoto et al. [15]. The child had 2 variants found on targeted exome testing NM_001287.5: (*CLCN7*):c.1505G > A:p. (Cys502Tyr) and NM_001287.5: (CLCN7): c.1729G > A: p. (Val577Met), both being missense variants.

In case 7 in the present report, a novel homozygous variant of unknown significance (VUS) *TCIRG1*:c.674G > A:p. (Gly225Asp) was identified. Mutations in this gene account for almost 50% patients of the severe osteopetrosis [13]. Genetic consultation and advice for prenatal diagnosis of early onset malignant osteopetrosis early in pregnancy is important for prevention.

**Figure 4:**
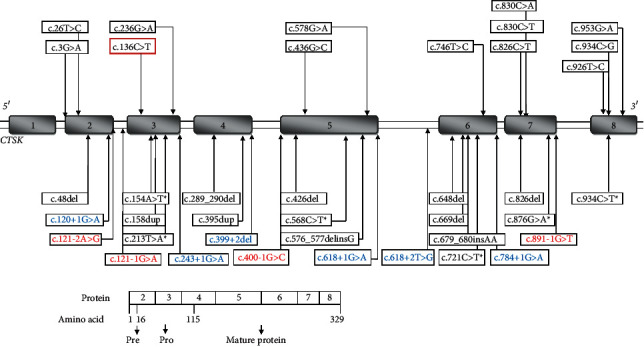
Pathogenic and likely pathogenic variants reported in *CTSK* gene leading to pycnodysostosis and sometimes osteopetrosis. The schematic representation of genomic structure of *CTSK* gene showing a total of 39 reported pathogenic (7) and likely pathogenic (32) gene variants for pycnodysostosis in 8 exons in which exon1 is non-coding (shown in shaded boxes). The top half of the figure show distribution of 13 missense variants in upper panel, 10 frameshift, 6 nonsense (with ^*∗*^ sign), 6 and 4 splice donor (blue colored) and splice acceptor (red colored) variants respectively in lower panel. The bottom half of the figure shows protein having 15 residues pre-region, 99 residues pro-region and 215 amino acid long mature proteins. The index child in present report showed variant c.136C > T (highlighted in red box) associated with pycnodysostosis.

Another bone disorder: pycnodysostosis is a relatively benign condition with later presentation, in which dense bones and acroosteolysis are characteristic features. The child in present report also had delayed bone age. A second mutation in the promoter region of *CTSK* cannot be ruled out in this case. The availability of NGS, along with traditional Sanger sequencing, has enabled better characterization of the high bone density disorders [16–18]. Variants described in *CTSK* gene are listed in Figure 4. Interestingly, variants in *CTSK* have been identified in early onset osteopetrosis in a previous study [5]. We also found one child with a clinical phenotype of osteopetrosis but homozygous variants in *CTSK* gene. Our child had hepatosplenomegaly and typical osteopetrosis phenotype. The variant *CTSK:* c.136C > T:p. (Arg46Trp) has been described earlier in ClinVar as likely pathogenic (ClinVar accession ID: VCV000553560.1). There is variable phenotype with several *CLCN7* and *CTSK* variants. It is possible that other genomic or epigenetic alterations have a role in causation of the final phenotype.

Thus, we found *CLCN7* and *CTSK* gene variants leading to high bone density phenotypes in children. High bone mass (HBM) results from effective coupling between factors involved in osteogenesis with those factors promoting angiogenesis. Recently, lncRNAs have been implicated in skeletal disorders with HBM [19]. Treatment strategies would involve promoting HBM in patients with osteoporosis and decreasing the bone mass using various analogs in those with osteosclerosis or osteopetrosis. Further studies will help unravel additional alterations and genotype-phenotype correlations.

## Data Availability

Data of patients who underwent NGS are available from the corresponding author.

## References

[1] Albers-Schönberg H. E. (1904). Röntgenbilder einer seltenen Knockenerkrankung. *Munchener Medizinische Wochenschrift*.

[2] Hall C. M. (2002). International nosology and classification of constitutional disorders of bone (2001). *American Journal of Medical Genetics*.

[3] Droz-Desprez D., Azou C., Bordigoni P., Bonnaure-Mallet M. (1992). Infantile osteopetrosis: a case report on dental findings. *Journal of Oral Pathology & Medicine*.

[4] Coudert A. E., de Vernejoul M. C., Muraca M., Del Fattore A. (2015). Osteopetrosis and its relevance for the discovery of new functions associated with the skeleton. *The Internet Journal of Endocrinology*.

[5] Pangrazio A., Puddu A., Oppo M. (2014). Exome sequencing identifies CTSK mutations in patients originally diagnosed as intermediate osteopetrosis. *Bone*.

[6] Stark Z., Savarirayan R. (2009). Osteopetrosis. *Orphanet Journal of Rare Diseases*.

[7] Tsuji Y., Ito S., Isoda T. (2005). Successful nonmyeloablative cord blood transplantation for an infant with malignant infantile osteopetrosis. *Journal of Pediatric Hematology*.

[8] Whyte M. P., Royce P. M., SteinmanB (2002). Osteopetrosis. *Connective Tissue and its Heritable Disorders: Medical, Genetic, and Molecular Aspects*.

[9] Subramaniam A., Singh A., Chavan M., Kunte S. (2008). Autosomal recessive osteopetrosis: case report of two siblings. *Oral Radiology*.

[10] Venkateshwar V., Vaidya A., Roy P., Sampat S., De J. (2003). Osteopetrosis. *Medical Journal Armed Forces India*.

[11] Phadke S. R., Gupta A., Pahi J., Pandey A., Gautam P., Agarwal S. S. (1999). Malignant recessive osteopetrosis. *Indian Pediatrics*.

[12] Cleiren E., Benichou O., van Hul E., Gram J., Bollerslev J., Singer F. R. (2001). Albers-Schonberg disease (autosomal dominant osteopetrosis, type II) results from mutations in the ClCN7 chloride channel gene. *Human Molecular Genetics*.

[13] Sobacchi C., Frattini A., Orchard P., Porraz O., Tezcan I., Andolina M. (2001). The Mutational spectrum of human malignant autosomal recessive osteopetrosis. *Human Molecular Genetics*.

[14] Li L., Lv S. S., Wang C., Yue H., Zhang Z. L. (2019). Novel CLCN7 mutations cause autosomal dominant osteopetrosis type II and intermediate autosomal recessive osteopetrosis. *Molecular Medicine Reports*.

[15] Okamoto N., Kohmoto T., Naruto T., Masuda K., Komori T., Imoto I. (2017). Novel ClCN7 compound heterozygous mutations in intermediate autosomal recessive osteopetrosis. *Human Genome Variation*.

[16] Hou W.-S., Brömme D., Zhao Y. (1999). Characterization of novel cathepsin K mutations in the pro and mature polypeptide regions causing pycnodysostosis. *Journal of Clinical Investigation*.

[17] Mandal K., Ray S., Saxena D. (2016). Pycnodysostosis: mutation spectrum in five unrelated Indian children. *Clinical Dysmorphology*.

[18] Khan B., Ahmed Z., Ahmad W. (2010). A novel missense mutation in cathepsin K (CTSK) gene in a consanguineous Pakistani family with pycnodysostosis. *Journal of Investigative Medicine*.

[19] Yang M., Guo Q., Peng H. (2019). Krüppel-like factor 3 inhibition by mutated lncRNA Reg1cp results in human high bone mass syndrome. *Journal of Experimental Medicine*.

